# Magnetic resonance imaging for individual prediction of treatment response in major depressive disorder: a systematic review and meta-analysis

**DOI:** 10.1038/s41398-021-01286-x

**Published:** 2021-03-15

**Authors:** Sem E. Cohen, Jasper B. Zantvoord, Babet N. Wezenberg, Claudi L. H. Bockting, Guido A. van Wingen

**Affiliations:** 1 Department of Psychiatry, Amsterdam Neuroscience, Amsterdam UMC, University of Amsterdam, Amsterdam, The Netherlands; 2 Department of Child and Adolescent Psychiatry, Amsterdam University Medical Center, Amsterdam, The Netherlands; 3 Centre for Urban Mental Health, Amsterdam University Medical Center, Amsterdam, The Netherlands

**Keywords:** Predictive markers, Neuroscience, Depression

## Abstract

No tools are currently available to predict whether a patient suffering from major depressive disorder (MDD) will respond to a certain treatment. Machine learning analysis of magnetic resonance imaging (MRI) data has shown potential in predicting response for individual patients, which may enable personalized treatment decisions and increase treatment efficacy. Here, we evaluated the accuracy of MRI-guided response prediction in MDD. We conducted a systematic review and meta-analysis of all studies using MRI to predict single-subject response to antidepressant treatment in patients with MDD. Classification performance was calculated using a bivariate model and expressed as area under the curve, sensitivity, and specificity. In addition, we analyzed differences in classification performance between different interventions and MRI modalities. Meta-analysis of 22 samples including 957 patients showed an overall area under the bivariate summary receiver operating curve of 0.84 (95% CI 0.81–0.87), sensitivity of 77% (95% CI 71–82), and specificity of 79% (95% CI 73–84). Although classification performance was higher for electroconvulsive therapy outcome prediction (*n* = 285, 80% sensitivity, 83% specificity) than medication outcome prediction (*n* = 283, 75% sensitivity, 72% specificity), there was no significant difference in classification performance between treatments or MRI modalities. Prediction of treatment response using machine learning analysis of MRI data is promising but should not yet be implemented into clinical practice. Future studies with more generalizable samples and external validation are needed to establish the potential of MRI to realize individualized patient care in MDD.

## Introduction

Major depressive disorder (MDD) is a debilitating disease, accounting for 40% of the global disability-adjusted life years caused by psychiatric disorders^1^. Depression is associated with impaired social functioning and unemployment and is associated with a wide range of chronic physical illnesses, such as diabetes and cardiovascular disease^2,3^. MDD is estimated to have a life-time prevalence of 20.6% in the United States^4^. Despite general consensus that effective treatment of depression is paramount for both a patient’s health and for reducing global burden of disease, global disease burden by MDD has not decreased in the past decades^5^. This is partly because treatment selection is based on trial and error, with no possibility to predict an individual’s response to a certain treatment^6^. Non-response to initial pharmacological and psychotherapeutic interventions is highly prevalent, with treatment-resistant depression affecting 20–30% of depressed patients in the current clinical practice^7–9^. Treatment of choice for patients who have not responded to pharmacological and psychotherapeutic treatments is electroconvulsive therapy (ECT), which produces remission in about 50% of therapy-resistant patients^10,11^. Furthermore, non-response can only be determined at least 4 weeks after initiation of pharmacotherapy, ECT requires 4–6 weeks on average, and effects of psychotherapy can even take 16 weeks to manifest^7,12^. Consequently, patients are regularly exposed to multiple failed treatments and might spend months to years waiting for successful treatment. This stresses the need for markers, which, before treatment commencement, can inform clinicians on the chance of responding to a particular treatment.

A large number of studies have correlated baseline clinical characteristics and biomarkers with MDD status and treatment outcome and have identified many factors that are associated with treatment success^13^. However, such descriptive analyses only provide inference at the group level and not at the level of the individual patient, which is required for clinical decision-making^14^. More recent studies have started to use machine learning analyses that aim to develop predictive models and that are tested using independent data^15^. More than with correlational analysis, single-subject response prediction studies using machine learning might be able to redeem the promise of individualized psychiatry^16^. Without being explicitly pre-programmed, these algorithms (either linear or non-linear) are able to learn from aggregated data in a patient sample using multivariate pattern recognition, in order to provide the best prediction of an output variable^17,18^. In predictive modeling, machine learning could enable clinicians to judge the viability of treatments for individual patients. As such, it might increase treatment efficacy, decrease illness duration, and reduce MDD’s impact on the global burden of disease.

Multiple modalities have been considered for single-subject response prediction. A recent meta-analysis covering different markers found neuroimaging to overall be most successful in predicting treatment response in depressed patients (i.e., more than phenomenological or genetic studies)^19^. However, the review pooled different treatments and neuroimaging modalities such as electroencephalography (EEG) and magnetic resonance imaging (MRI). Since it did not differentiate between prediction success in different neuroimaging techniques, the study offers little insight into treatment-specific biomarkers or specific (MRI) modalities. A recent meta-analysis on EEG for individual prediction of antidepressant treatment response found reasonable accuracy (72% sensitivity and 68% specificity) but concludes that EEG should not yet be used clinically as a prediction tool, since generalizability and validity of the reported studies are limited^20^. However, a meta-analysis of prediction accuracy in anti-depressive treatment that specifically focuses on MRI does not yet exist, which may reveal a better predictive value than EEG.

The primary aim of the present study was to calculate the aggregate classification performance of predictive MRI biomarkers in patients with MDD using a bivariate random-effect model meta-analysis. We further investigated whether classification performance was influenced by intervention type (i.e., pharmacotherapy, psychotherapy, or ECT) or imaging modality (i.e., structural MRI (sMRI), resting-state functional MRI (fMRI), task-based fMRI, diffusion tensor imaging (DTI)).

## Methods and materials

### Inclusion and exclusion criteria

Two authors (S.E.C. and B.N.W.) included studies using any form of MRI (structural, resting-state, task-based, spectroscopy, DTI), which were conducted at baseline, i.e., within 4 weeks before the start of antidepressant treatment. Furthermore, inclusion criteria were an overarching definition of antidepressant treatment according to the current NICE guidelines and a non-selective patient population with MDD suffering from a current depressive episode. Studies that used feature selection based on in-sample data without validating prediction outcomes either internally (e.g., through cross-validation) or externally (through independent set validation) were excluded. Inclusion or exclusion conflicts were resolved by consensus or if necessary by authors J.B.Z. and G.A.v.W.

### Search strategy

We conducted a search in EMBASE, Medline, PsycInfo, and Web of Science databases. Each database was searched from inception to January 2020. Furthermore, we searched the WHO International Clinical Trial Registry Platforms search portal for registered and unpublished studies, and we looked for “gray” literature such as abstracts and conference articles through conference websites and from other relevant sources. Additionally, we checked included articles for references and conducted citation screening. For a full account of our search strategy and inclusion criteria, see the Supplementary Material.

### Data extraction

Two authors (S.E.C. and B.N.W.) independently extracted data from included studies, including the number of participants, patient population and depression severity subtype, treatment history, antidepressant intervention and outcome measures, response/remission rates, neuroimaging technique, brain region and feature selection, method of analysis, and validation strategy (see Table 1). From the included articles, we extracted the confusion table (a 2 × 2 table for correctly and incorrectly classified patients) for sensitivity or specificity. If these were not supplied, we computed the matrix from additional information in the article. If multiple studies analyzed the same patient sample, we used mean outcome measures based on these studies. If necessary, we contacted authors requesting additional information.

**Table 1:** Methodological summary of the studies.

Study + year	*n*	Outcome	Intervention	Duration	Modality	Analysis	Validation
Costafreda et al. 2009—1	16	Remission	CBT	16 wk	tbfMRI	SVM	LOO CV
Siegle et al. 2012	12	Response	CBT	12 wk	tbfMRI	RF	Ind. replication
Queirazza et al. 2019	37	Response	CBT	6–10 wk	tbfMRI	SVM, LR	LOO nested CV
Van Waarde et al. 2015	45	Remission	ECT	10 wk	rsfMRI	lSVM	LOO CV
Moreno-Ortega et al. 2019	19	Remission	ECT	ns	rsfMRI	LR	LOO CV
Sun et al. 2019	122	Remission + remission	ECT	3–4 wk	rsfMRI	LR	LOO CV
Redlich et al. 2016	23	Response	ECT	3–8 wk	sMRI	lSVM/GPC	LOO CV
Wade et al. 2016	34	Response	ECT	2–7 wk	sMRI	RBFSVM	LOO CV
Cao et al. 2018	24	Response + remission	ECT	3–4 wk	sMRI	lSVM	LOO CV
Jiang et al. 2018	38	Remission	ECT	3–4 wk	sMRI	LR	10-fold LOO CV+ Independent cohort rep
Wade et al. 2017 Leaver et al. 2017	44^a^	Remission Response	ECT ECT	ns ns	sMRI rsfMRI, aslMRI	RF RBFSVM	Nested CV 5-fold LOO CV
Drysdale et al. 2017	124 30	Response	rTMS	4–6 wk	rsfMRI	lSVM	LOO CV Ind. replication
Cash et al. 2019	33	Remission	rTMS	5–8 wk	rsfMRI	lSVM	LOO + *k* fold CV
Costafreda et al. 2009—2 Nouretdinov et al. 2011	18^a^	Remission Remission	SSRI SSRI	8 wk 8 wk	sMRI sMRI	lSVM TCP	LOO CV LOO CV
Gong et al. 2011	46	Response	SSRI/TCA/SNRI	12 wk	sMRI	lSVM	LOO CV
Marquand et al. 2008	20	Response	SSRI	8 wk	tbfMRI	lSVM	LOO CV
Godlewska et al. 2018	32	Response	SSRI	6 wk	tbfMRI	LR	LOO CV
Meyer et al. 2019	22	Remission/non-response	SSRI	8 wk	tbfMRI	LR	LOO CV
Karim et al. 2018	49	Remission	SNRI	12 wk	tbfMRI	LR	10-fold LOO CV
Patel et al. 2015	19	Remission	SSRI/SNRI	ns	rsfMRI, DTI, sMRI	ADTree/lSVM/RBFSVM/L1LR	Nested LOO CV
*iSPOT trials*	77^a^		SSRI/SNRI	8 wk			
Korgaonkar et al. 2014		Remission			DTI	LR	*K*-fold CV
Williams et al. 2015		Response			tbfMRI	LDA	LOO CV
Goldstein-Piekarski et al. 2016		Remission			tbfMRI	LR	10-fold LOO CV
Grieve et al. 2016		Non-remission			DTI	LR	Independent rep
Goldstein-Piekarski et al. 2018		Remission			rsfMRI	LR	LOO CV

Reported sample sizes were not necessarily equal in articles with overlapping sample.

*SSRI* selective serotonin reuptake inhibitor, *TCA* tricyclic antidepressant, *SNRI* serotonin-norepinephrine reuptake inhibitor, *ECT* electroconvulsive therapy, *CBT* cognitive behavioral therapy, *rTMS* repetitive transcranial magnetic stimulation, *iTBS* intermittent theta burst stimulation, *AP* antipsychotics, *ns* not specified, *tb* task based, *rs* resting state, *asl* arterial spin labeling, *fMRI* functional magnetic resonance imaging, *sMRI* structural magnetic resonance imaging, *WB* whole brain, *ROI* region of interest, *DTI* diffusion tensor imaging, *lSVM* linear support vector machine, *RBF* radial basic function, *TCP* transductive conformal predictor, *LR* logistic regression, *LinR* linear regression, *LDA* linear discriminant analysis, *RF* random forest, *LOO CV* leave-one-out cross-validation, *wm* white matter, *sLR* stepwise linear regression, *beta-w* beta-weights, *LARS* least-angle regression, *PMVD* proportional marginal decomposition.

^a^*n* is a weighted average across studies.

### Meta-analytic method

For quantitative analysis, we used confusion matrices to pool studies using Reitsma’s bivariate random effect model, as suggested in the Cochrane handbook for diagnostic tests of accuracy studies^21,22^. We used this method for computing our main outcomes, which were the overall area under the summary receiver operating characteristic (SROC) curve, sensitivity, and specificity, as well as sensitivity and specificity of intervention subsets. Additionally, we performed a separate bivariate regression for modalities (fMRI and sMRI) by including from each study both sMRI and fMRI, if provided in the original article or after our request for further information. As a post hoc analysis, we excluded DTI from this regression, and in the fMRI group, we subdivided resting-state and task-based modalities.

### Heterogeneity and publication bias

To visualize between-study differences, we conducted a univariate random-effect forest plot of the diagnostic odds ratios (ORs), subdivided per treatment group. We identified clinical and statistical heterogeneity by visually assessing confidence interval (CI) overlap and by identifying outlying studies. We avoided using an objective measure of heterogeneity, since these have shown to be inappropriately conservative for accuracy studies^23^. Rather, we used a random-effect model that assumes that our data was heterogeneous and set out to investigate potential sources of heterogeneity^22^. We did not perform any sensitivity analyses, as no studies were of such low quality, or were such outliers that sensitivity analysis was appropriate. To assess sample size effects and possible publication bias, we used Deeks’ test, as recommended for diagnostic accuracy studies^24,25^. For assessing quality of the primary studies, we used the QUADAS-2 tool^26^. We pre-specified methods in the PROSPERO database for systematic reviews (registration number CRD42019137497). All analyses were conducted using the mada and metafor package in R^27–29^.

## Results

### Search results

**Fig. 1:**
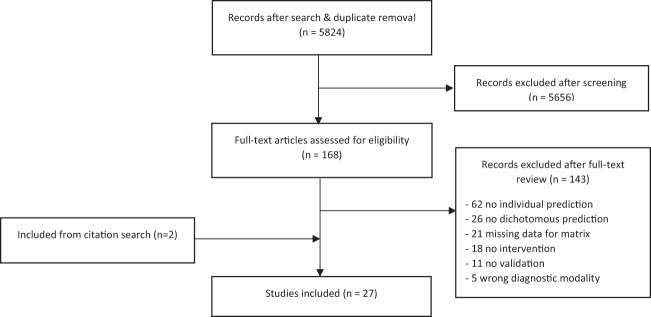
Flow diagram of the study inclusion process. *n* number.

Our search yielded 5824 hits, 168 of which were included for full-text review (see Fig. 1). After contacting the authors for additional information, we excluded 21 studies for not reporting data necessary for reconstructing a confusion matrix, all of which were “gray literature”, i.e., abstracts or conference summary articles. Furthermore, we excluded 11 articles for not reporting any form of validation of their prediction model. After exclusion of non-eligible studies and, through citation searching, addition of 2 eligible studies that did not come up in search hits, 27 remained^30–56^.

### Description of the study characteristics

We included 27 studies with an accumulated number of 957 unique patients and a mean sample size of 44 per study, with a median of 33 (see Table 1 for a full methodological study summary. Please refer to Supplementary Table 1 for an overview of patient characteristics and study demographics). Three patient samples were used in more than one article^30,32,40,41,51–55^.

Of the included studies, 50% used some form of pharmacotherapeutic intervention (total *n* = 283), all of which administered a clinically viable dosage, with response time varying from 2 weeks (early response) to 12 weeks. Only one study did not use selective serotonin reuptake inhibitors (SSRIs), instead using an serotonin-norepinephrine reuptake inhibitor (SNRI)^49^. Three studies used either an SSRI or SNRI, and one of these three chose a tricyclic antidepressant as a third treatment option^45,50,57^. ECT was administered in 35% of studies (total *n* = 285), 8% used transcranial magnetic stimulation, and 8% used cognitive therapy. Most studies used either sMRI (31%) or task-based fMRI (31%), most often using emotional stimuli, 19% used resting-state fMRI, and 8% used DTI. Two studies combined multiple modalities^40,50^.

As machine learning paradigm, 31% studies used support vector machine (SVM) for data-analysis, while 28% used logistic regression. After comparing classification accuracy with multiple algorithms (among others, SVM and random forest), Patel and colleagues used an alternating decision tree method^50^. For validation, 85% used leave-one-out cross-validation. Two studies used an independent cohort to validate their results, while one study first cross-validated classification results, after which authors validated their prediction model in two small, independent cohorts, achieving similar results^39,43,53^. For additional information on approaches to imaging analysis, please refer to Supplementary Table 2.

### Meta-analysis

#### General outcome

**Fig. 2:**
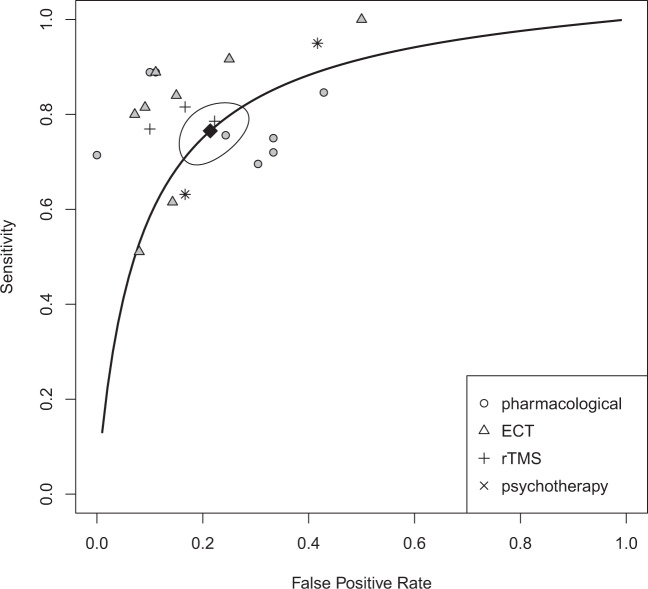
Overall accuracy measures: area under the curve 0.84 (95% CI 0.81–0.87), sensitivity 77% (95% CI 71–82), specificity 79% (95% CI 73–84). Reitsma bivariate SROC model of the receiver operating characteristic curve. Summary of sensitivity and false-positive rate (1 − specificity) is indicated in black, sensitivity and false-positive rates for different interventions are gray-scale. ECT electroconvulsive therapy, rTMS repetitive transcranial magnetic stimulation, pharmacological pharmacotherapeutic antidepressive interventions.

After pooling results from studies with overlapping patient samples, we quantitatively analyzed 22 samples, including one independent cohort replication that we have interpreted as a separate study^43^. For all imaging modalities and interventions taken together, the meta-analytic estimate for the SROC AUC was 0.84 (95% CI 0.81–0.87), with 77% sensitivity (95% CI 71–82) and 79% specificity (95% CI 73–84), amounting to a moderately high classification performance (see Fig. 2).

#### Intervention differences

Sensitivity and specificity of ECT interventions were 80% (95% CI 73–85) and 83% (95% CI 72–90), respectively, compared to 75% (95% CI 68–82) and 72% (95% CI 64–80) for antidepressant medication. Exclusion of the studies that did not use SSRI as pharmacological agent had little influence on the results^49^. Although prediction outcomes in ECT studies do show a trend toward higher precision, CIs overlapped (see Table 2). With only few primary studies, sensitivity and specificity for psychotherapy were, respectively, 84% (95% CI 68–92) and 72% (39–92), for repetitive transcranial magnetic stimulation (rTMS), respectively, 79% (95% CI 71–86) and 82% (74–88).

**Table 2:** Summary estimates of sensitivity/specificity for different interventions.

Intervention group	Sensitivity	95% CI	Specificity	95% CI
Combined	77%	71–82	79%	73–84
Medication	75%	68–82	73%	64–80
ECT	80%	73–85	83%	72–90
Psychotherapy	84%	68–92	72%	39–92
rTMS	79%	71–86	82%	74–88

*CI* confidence interval, *rTMS* repetitive transcranial magnetic stimulation, *ECT* electroconvulsive therapy.

#### Modality differences

In order to assess whether sMRI studies yielded different performance measures compared to fMRI studies, we performed random-effect meta-regression for modality subtypes. When comparing fMRI and sMRI, *z*-regression values for sensitivities and specificities were non-significant, suggesting that prediction success for structural or functional neuroimaging did not differ between studies (see Table 3). Post hoc analysis excluding DTI and subdividing task-based and resting-state fMRI did not alter the results.

**Table 3:** Bivariate random-effect meta-regression *z*-scores for modality as covariate.

	Point estimate	Standard error	95% Lower	95% Upper	*z*-value	*p* Value
Sensitivity	0.221	0.233	−0.236	0.677	0.948	0.343
Specificity	0.217	0.252	−0.77	0.711	0.861	0.389

*p* Values for both sensitivity and specificity >0.05, i.e., *z*-score differences for functional and structural MRI are non-significant.

### Quality assessment

Three studies included only late-life depression, which reduces applicability in the general MDD population (see Supplementary Fig. 1 and Supplementary Table 3). In terms of flow and timing, drop-outs were a common issue, with 10 studies having a drop-out rate of ≥30%, while 11 studies did not clarify drop-outs, possibly leading to attrition bias. Furthermore, two studies adapted the definition of response to create an even split in responders/non-responders, causing applicability concerns^45,48^. One study did not pre-specify the pharmacological intervention^50^.

### Heterogeneity and publication bias

**Fig. 3:**
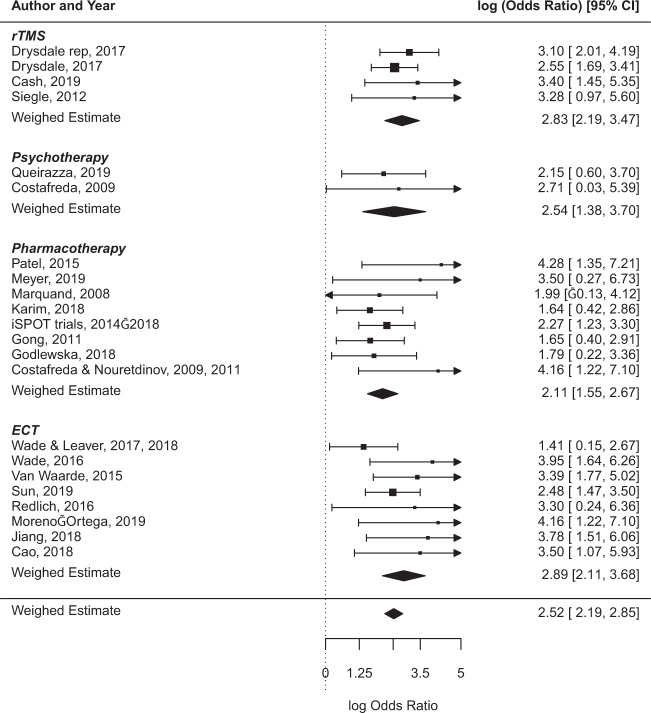
Univariate random-effect forest plot of natural logarithm of diagnostic odds ratios. Summary estimates for odds ratios are computed assuming normal distribution. CI confidence interval, rTMS repetitive transcranial magnetic stimulation, ECT electroconvulsive therapy.

The univariate forest plot of diagnostic performance (in ln OR) showed considerable overlap in CIs between studies with different ORs, indicating that heterogeneity might be caused by sample variance (see Fig. 3)^23^. As described in the study description above, inter-study differences were present in population, modalities, intervention type, response/remission definition, feature selection, and analysis technique. Deeks’ funnel plot asymmetry test showed study size and diagnostic OR to be inversely related (*p* = 0.044; see Supplementary Fig. 2), indicating that classification performance was lower in studies with larger samples. Inspection of the gray literature that was excluded due to missing information in order to construct a confusion matrix (all of which were conference/poster abstracts) showed that the gray literature had comparable mean sample sizes (*n* = 22, mean *n* = 56) and accuracies (ranging from 73 to 95%) compared to the included studies. For an overview of gray literature results, see Supplementary Table 4.

## Discussion

Our results show that machine learning analysis of MRI data can predict antidepressive treatment success with an AUC of 0.84, 77% sensitivity, and 79% specificity (Fig. 2). Furthermore, we did not find a difference in classification performance between studies using pharmacotherapy and ECT. Although ECT showed somewhat higher sensitivity and specificity, CIs largely overlapped between the two intervention types (Table 2). There were few primary studies for psychotherapy and rTMS, which also show overlapping CIs. In addition, classification performance of sMRI and fMRI did not differ significantly (Table 3).

To our knowledge, this is the first meta-analysis specifically examining MRI for predicting treatment effects in depression. The overall classification performance is comparable to the one reported by Lee et al., who found a general accuracy of 85% when combining the results for different neuroimaging modalities (defined as EEG, computed tomography, positron emission tomography, or MRI)^58^. Those results were, however, based on a total of 8 MRI studies, whereas our search resulted in 22 individual studies for analysis. This is partly due to the time gap between studies, which underscores the rapid development in this research area. Our results show that MRI prediction studies perform somewhat better than EEG (AUC of 0.76) and comparable to accuracy of *diagnostic* classification studies with MRI that distinguishes depressed patients and healthy controls^20,59^. In contrast to the review of EEG studies, we excluded studies that tested their model on the training set, which increased generalizability of our sample and avoided presenting inflated accuracy results.

Clinical practice would require different prediction approaches for a broad range of specific settings. It would be useful to have a single predictive test for therapy-resistant patients, especially to guide decision-making for invasive treatments such as ECT. For example, ECT is associated with cognitive side effects that are preferably avoided in case the treatment is unsuccessful^60^. In addition, ECT is only applied in 1–2% of patients with persistent or severe depression and a biomarker that indicates a high probability of success may reduce the hesitance of its use^61^. However, for most treatments, a differential biomarker would be preferable, which would enable selecting the treatment with the highest chance of success. As of yet, no MRI study has used such prospective prediction and subsequent treatment matching to guide decision-making between two treatment options (for instance, between cognitive behavioral therapy and an SSRI). Furthermore, no studies have yet compared efficacy of prediction-guided treatments versus regular treatment based on patient–clinician preference. Thus, although the predictive performance of MRI biomarkers is certainly promising, the current study designs do not yet enable the translation of research findings to the clinic.

Generally, studies were of acceptable quality, although drop-out rates could cause concern in terms of reliability. Drop-out rates were not mentioned in 11 studies, and for 10 studies, drop-out rates were >30% without using an intention-to-diagnose approach. Not accounting for drop-outs, who might be less likely to respond to treatment, could inflate response/remission data and consequently alter sensitivity and specificity of the predictive test. Additionally, our results show between-study variety regarding the response criterion, which typically consisted of clinical response (≥50% symptom reduction) or symptom remission. Different clinical settings might require different prediction outcomes. For instance, one could expect treatment of a first-time depressive episode to lead to complete remission, while in severe treatment-resistant depression, response might be a more practical and achievable goal^62^. Authors should take care to pre-specify which outcome they will use and why that outcome is the most appropriate for their sample or intervention.

Furthermore, although no objective investigation for clinical heterogeneity in prediction studies exists, our random-effect forest plot shows considerable overlap of CIs with differing study results, implying the presence of sampling variation (Fig. 3)^22^. Clinical variance between samples is an important obstacle in generalizability of any diagnostic or predictive marker, especially in psychiatric illnesses such as MDDs, which is heterogeneous in both its clinical and neurophysiological manifestation^63,64^. Thus, inter-sample diversity of inclusion criteria and methodological design might hamper the realization of a reliable predictive biomarker.

In the current literature on diagnostic accuracy studies, the possibility of publication selection as a source of bias is still under debate^25,65^. Common forms of formalizations of publication bias, such as the Egger’s or Begg’s test, are not recommended for meta-analyses of prediction studies, since their sensitivity in diagnostic accuracy studies is generally poor^23^. However, the recommended Deeks’ funnel plot asymmetry test (see Supplementary Fig. 2) shows the presence of a sample size effect, with the *n* of a study being negatively correlated to classification performance, which could be attributable to publication bias^66^. Another explanation of this significant correlation might be that large-scale studies with large samples are more likely to consist of heterogeneous patient groups, which in turn reduces prediction accuracy^67^. As a further exploration of publication bias, our search also took into account gray literature, which indicated that publication (or positive result) bias was absent. In conclusion, quantitative testing could not distinguish between a real effect (due to accuracy reduction in large heterogeneous samples) or publication bias. Although the gray literature deems its presence less likely, we cannot exclude the presence of publication bias.

The following limitations warrant further discussion. First, we did not find modality differences, but studies conducting fMRI research might have also attempted prediction with (less time-consuming and cheaper) sMRI, which remained unpublished. Although we did contact authors for additional information, response was poor, so we were unable to rule out reporting bias for modality differences. We would advise authors of future studies to publish non-significant results as well as significant but less accurate results, since both are potentially useful in comparing the merits of different modalities. Second, the number of studies predicting the effects of psychotherapy, specifically cognitive therapy, outcome was low, resulting in a blind spot for a commonly deployed treatment in MDD^68^. Third, cross-validation in small samples results in large variation of the estimated accuracy, and as indicated above, accuracy reduces with larger sample heterogeneity^67,69^. Since the mean sample size of our studies was 44 (with a median *n* of 33), the reported results may be optimistic because of overfitting. Overfitting is a cause for concern specifically in MRI studies, with relatively small sample sizes and large amounts of fitted data^70^. Furthermore, characteristics of the test set during cross-validation will approximate the characteristics of the training set more than when tested in the general population, due to selection bias^71^. Only two included studies replicated their training data in an independent cohort, and one included study used an out-of-sample cohort to further test their cross-validated results, leaving the question open to which extent the majority of results can be generalized to new patients.

In order to optimize patient care, reduce treatment resistance, and shorten duration of illness, developing models that predict treatment success on individual-patient level is an urgent task. In a 2012 consensus report on diagnostic imaging markers in psychiatry, the American Psychiatric Association research council proposed 80% sensitivity and specificity as prerequisite for the clinical application of a biomarker^72^. Furthermore, biomarkers should be ideally be reliable, reproducible, non-invasive, simple to perform, and inexpensive. The results for an ECT biomarker fulfilled the 80% criterion, but the results for a medication biomarker fell short. But following these terms, primarily reproducibility has not yet been sufficiently well established with small sample sizes and external validation in only a minority of studies. This precludes recommending MRI for treatment response prediction in clinical practice at this point. Future multicenter studies with large patient samples that represent clinical heterogeneity are required to warrant MRI biomarker generalizability^73^. However, one might question whether excellent generalizability is a goal that should be aimed for: if each clinical site were to develop its own locally reliable and replicable biomarker that incorporates the local hardware, patient, and treatment variability, the predictive accuracy is expected to be higher than when all potential sources of heterogeneity are accounted for^67,74^. Standard machine learning analysis would, then, mean a departure from the traditional universalist paradigm in diagnostics and instead initiate a shift to a paradigm of localization: heterogeneous yet locally applicable classification models. This will enable to retrain predictive models to obtain even better performance with more data after biomarker deployment. And this may enable to take advantage rather than disadvantage from (inevitable) hardware upgrades, such as higher signal-to-noise for new generations of MR scanners and coils.

In conclusion, prediction of treatment success using machine learning analysis of MRI data holds promise but has not transcended the research status and should not yet be implemented into clinical practice. Once it overcomes the aforementioned hurdles, MRI may become a clinical decision support tool aimed to reduce unsuccessful treatments and improve treatment efficacy and efficiency.

## Supplementary information

Supplemental material
