# Supplementary material for: Magnetic resonance imaging for individual prediction of treatment response in major depressive disorder: a systematic review and meta-analysis
Source: Transl Psychiatry. 2021 Mar 15;11:168. doi: 10.1038/s41398-021-01286-x (PMC7960732; doi:10.1038/s41398-021-01286-x)
Supplement: Supplementary file 1 — Supplemental material [file 41398_2021_1286_MOESM1_ESM.pdf]

Supplementary material

Supplementary methods

Search Strategy

Our search strategy included terms regarding the population, the diagnostic predictor and the study results, in a measure of accuracy. Studies were included up to January 2020. We used no language or date and we excluded animal/non-human studies. The search was conducted by a clinical librarian and search specialist (JD), in order to ensure a high degree of thoroughness.

We searched the following electronic databases: EMBASE, Medline and PsycInfo and Web of Science. Our Medline search was constructed as follows:

((depression/ or postnatal depression/ or major depression/ or treatment resistant depression/) OR (mdd or major depressi* or unipolar or postnatal depressi* or post natal depressi* or postpartum depressi* or post partum depressi* or refractory depression or (depressi* adj3 resistan*) or late life depressi*).ab,kw,ti.)) AND

((nuclear magnetic resonance imaging/ or diffusion tensor imaging/ or exp neuroimaging/ or functional magnetic resonance imaging/) OR ((magnetic resonance or mr imaging or mri or fmri or dti or diffusion tensor or tensor imaging or structural neuroimaging or functional neuroimaging or structural neuranatomy or functional connectivity).ab,kw,ti.)) AND

(("sensitivity and specificity"/ or predictive value/ or exp diagnostic error/ or diagnostic accuracy/) OR (("sensitivity and specificity" or npv or ppv or roc or predict* or prognos* or accuracy).ab,kw,ti.))

Additionally, we checked every included article for relevant references. As of yet no central registration database exists for unpublished diagnostic studies, however we searched in the WHO International Clinical Trial Registry Platforms search portal for registered and unpublished studies. Furthermore, we looked for ‘grey’ literature such as (poster) abstracts and conference articles through conference websites (SOBP, ISAD, APAAM, ACNP, ECNP, HBP, WCP, Molecular Psychiatry, ADAA, going back to 2009) and from other relevant sources.

Our inclusion criteria, as well as our meta-analytic methods of procedure were pre-registered in the PROSPERO register of systematic reviews (registration ID CRD42019137497).

Inclusion and exclusion

Inclusion criteria:

- Adults (18 years or older) diagnosed with Major Depressive Disorder, as diagnosed using the DSM III, IV or 5 criteria. We included the entire MDD-population, including all severity subtypes. Therapy resistance status was allowed to range from naive to resistant. We included both in- and outpatients.
- Magnetic Resonance Imaging (structural MRI, task-based functional MRI, resting state functional MRI, diffusion tensor imaging) before the start of antidepressive treatment (all-compassing, ranging from psychotherapy to pharmaceutical treatment or electroconvulsive therapy, as included in the National Institute for Health and Care Excellence Guidelines for antidepressive treatment) [1]. To ensure that data used for treatment prediction correspond with the situation at treatment-baseline, MRI had to be performed within a month before treatment commencement date.
- Accuracy of prediction had to be evaluated by comparing the predicted outcome, to a validated disease-severity questionnaire or semi-structures interview *after* treatment. Such questionnaires/interviews included: the Hamilton Depression Rating Scale -17, the Beck Depression

Inventory, the Montgomery Asberg Depression Rating Scale or the (Quick) Inventory of Depressive Symptomatology, specified as either self-rated or clinician rated. We considered the validity of all these rating scales to be equal. Furthermore, we included studies that measure symptom severity/response status within 12 weeks after treatment commencement. We chose this cut-off to allow pharmacotherapeutic dose escalation corresponding with routine clinical practice, and to allow time for psychotherapeutic therapies to take effect.

- We included studies that set out to predict, on the level of the individual patient with a depressive episode, response or remission to therapy. We did allow for any therapy that is part of official treatment guidelines, since our main aim is to investigate the general possibility of MRI to predict therapeutic response. We allowed for psychiatric co-medication such as benzodiazepines, since these are administered routinely in daily clinical practice.
- We included any study with a pre-specified definition of response/remission.
- We allowed studies to have defined prediction as sensitivity/specificity, positive or negative likelihood ratio, positive or negative predictive value, a measure of overall accuracy, the area under the ROC-curve, a Youden’s index, diagnostic odds ratio or any other measure that illustrates accuracy and/or may be used to compute a confusion matrix.

Exclusion criteria:

- We excluded studies with patients younger than 18 years old, or patients with a bipolar disorder. We chose to exclude patients with a depressive episode in context of a bipolar disorder, since there is evidence that bipolar depression exhibits characteristics which significantly differ from unipolar depression, both clinically and neurobiologically [2, 3]. If an article included a mixed sample of bi- and unipolar depressant patients, we excluded the study if more than two-thirds of patients suffered from bipolar disorder

- If studies used feature selection based on in-sample data, we excluded them if they did not validate their prediction outcome either internally (e.g. through cross-validation) or externally (through independent set validation). We chose this approach since accuracy measures based on data that are included in model training are positively biased and have unknown (and presumably low) levels of generalizability, deeming them irrelevant for making a substantiated recommendation, as was the goal of our review. Studies that did not validate their data, but did use a-priori defined methods and features for analysis, were included.

Data extraction

Two authors (SC and BW) extracted, if provided, from included studies the following data: number of participants, gender distributions (male/female), age, mean severity pre-intervention, number of prior episodes, duration of current (index) depressed episode, whether or not patients had a history of failed antidepressant treatment, if patients used current psychiatric medication (and if so, which ones), specific exclusion criteria. Furthermore, we specified treatment, dosage/frequency, duration of treatment, defined endpoint and response or remission rates. For the diagnostic tests, we specified modality, region of interest, machine-learning algorithm, cross-validation analysis. As results, we extracted sensitivity, specificity and the confusion matrix (true prositive, false negative, false positive, true negative). For the full result table, please refer to **supplementary table 1.**

Quality analysis

For risk of bias in patient selection we asked if a consecutive or random sample of patients enrolled and if the study used proper exclusion criteria. For the index test (i.e. the MRI biomarker) we asked if multiple comparison testing was used for feature selection. For the reference standard (i.e. the questionnaire or rating scale) we asked if the reference standard was validated for MDD. For flow and timing, we considered an appropriate interval between MRI and questionnaire (i.e. within 12 weeks),

and whether all patients did indeed receive the same rating scale. Furthermore, we considered which percentage of patients who underwent an initial predictive MRI, did indeed finish the treatment protocol. If there was a drop-out rate of more than 30%, we considered this as a high risk of bias. We added a section intervention, in which we took into account whether the intervention (kind, dosage, duration) was pre-specified at baseline.

Studies that selected patients who did not solely include MDD patients (i.e. also included a small portion of depressed bipolar patients) or who included a specific subsection of MDD patients, such as late-life depression, were registered as having a high applicability concern. Furthermore, if the index test (i.e. the MRI) were to be combined with clinical data other than age, disease severity and sex, we considered concerns to be 'high'. Cut-offs of response/remission the reference standard (i.e. rating scale/questionnaire) were preferable pre-specified and if not, applicability concerns were high. If for the intervention, frequency, dosage and time did not match clinical standards, applicability concerns were high.

Meta-analytic procedure

We pooled studies using a bivariate random effect model according to Reitsma, as suggested in the Cochrane handbook for diagnostic tests of accuracy studies [4, 5]. Main outcomes were the overall area under the sROC-curve and sensitivity/specificity, as well as sens/spec of intervention subset (pharmacological treatment, specified in appropriate considering amount of studies, electroconvulsive therapy, psychotherapy, transcranial magnetic stimulation and any other intervention with a 5 or more studies). A cause of heterogeneity in diagnostic or predictive tests is the threshold effect; in prediction models, studies use varying cut-offs for classifying a patient as responder or remitter. This threshold effect causes an inverse relation between sensitivity and specificity. Most neuroimaging studies establish empirically which threshold should will used for prediction, based on which produces the

highest overall accuracy [66]. The bivariate sROC curve is specifically suitable for this kind of heterogeneity since its visual properties take the correlative relation between sensitivity and specificity into account [7]. For computing confidence intervals for the area under the ROC-curve, we used the method of Hanley and McNeil (1982).

To detect sample size effect and possible publication bias, we plotted a funnel plot based of 1/√ (effective sample size) as a function of the natural logarithm of the diagnostic odds ratio [8]. Known as the Deeks' test, this function is the recommended test for sample size effect [9]. More common forms of formalizations of publication bias, such as the Egger's or Begg's test are not recommended for this review, since their sensitivity for diagnostic accuracy studies is generally poor [5, 8 7].

QUADAS quality assessment

Risk of bias

1) Patient selection

- Was a consecutive or random sample of patients enrolled?

- Did the study avoid inappropriate exclusions?

2) Index test

- Were the index test results interpreted without knowledge of the results of the reference standard?

- If a threshold was used, was it pre-specified?

3) Reference standard

- Is the reference standard likely to correctly classify the target condition?

- Were the reference standard results interpreted without knowledge of the results of the index test?

4) Flow/timing

- Was there an appropriate interval between index test and reference standards?

- Did all patients receive a reference standard?

- Did patients receive the same reference standard?

- Were all patients included in the analysis?

5) Intervention

- Was the intervention (kind, dosage, duration) pre-specified?

<u>Applicability</u>

1) Risk of bias

- Is there concern that the included patients do not match the review question?

2) Index test

- Is there concern that the index test, its conduct, or interpretation differ from the review question?

3) Reference standard

- Is there concern that the target condition as defined by the reference standard does not match the review question?

Supplementary discussion

Discussion of quality assessment

Not one study used a consecutive patient enrollment, causing concern for selection bias and thus low applicability. A problem might arise specifically when researchers choose not to include certain patients for their study, on the basis of reasons outside of a-priori protocol. Such reasons might include expectations about whether patients will be reliable in finishing the study, co-morbid substance abuse or predominantly 'psychosocial' events leading up to depressive disorder. Consecutive enrollment is not an issue generally discussed within diagnostic studies, but since our included studies are at the same time intervention studies, these concerns might be especially relevant.

Furthermore, three studies included only late-life-depression, reducing applicability in the general MDD population, as late-life-depression, although being symptomatically similar with MDD in a lot of aspects, is thought to co-occur more often with medical disorders such as vascular illnesses and neurocognitive disorders.

In the terms of flow and timing, drop-out-rates, as discussed in the main body of text, cause issues around attrition bias. All studies used multiple correction control in feature selection for the index test, through the process of cross-validation, as was one of the inclusion criteria. One study did not pre-specify the pharmacological intervention (Patel et al.). Two studies adapted their definition of response to create an even split in responders/non-responders, causing applicability concerns since one would have to know exactly which predefined outcome to predict for response prediction to be clinically relevant (Meyer et al., 2019; Leaver et al., 2018).

Non-validated studies

Remarkably, we did exclude as much as eleven articles that did not use a form of validation after they used their training data for response prediction. In most of these cases, dichotomous response prediction

was a secondary, or post-hoc, analysis, and some authors do note that these results might not be generalizable or should be interpreted with caution. However, all authors did report their results in the abstract, clouding research on this topic. Furthermore, for a clinician, critically evaluating the absence of cross-validation or independent test set validation is infeasible which could lead to overestimation of prediction success. Therefore, we would advise against publishing non-validated prediction results if these studies use feature selection in the training sample.

Feature selection and analysis

We did not discuss different machine-learning methods or feature-selection techniques in depth, since these issues go beyond the scope of our research question. However, summary of our results (see **supplementary table 2**) indicates that no single region-of-interest, network-analysis or functional task seems to stand out in prediction success, although with our data we could not quantitatively substantiate this finding. For instance, one found an important predictive role for the subcallosal cingulate gyrus, while another, after whole-brain data mining, did not include this brain part in their final predictive model [10, 11]. Thus, cerebral processes which influence treatment success seem to be overdetermined and prediction might be made in a multitude of ways

Supplementary table 1, summary of patient characteristics

Study + yr	Sex (F)	Mean age (yr)	Location - setting	Severity	Medication history	Current medication	Diagnostics
Marquand, 2008	70%	43.7	UK, outpatient	HDRS-17 21.2	-	None, > 4 wk washout	DSM IV
Costafreda, 2009	78%	44.2	UK, outpatient	HDRS-17 20.6	-	None, 4 wk washout	DSM IV
Costafreda, 2009			UK, outpatient	HDRS-17 20.9	-		DSM IV
Nouretdinov, 2011	78%	44.2	UK, outpatient	HDRS-17 20.6	-	None, 4 wk washout	DSM IV
Gong, 2011	48%	39.8		HDRS-17 23.9	AD naive	none	
Siegle, 2012	84%	36.1	USA, outpatient	HDRS-14 20.3	-	None, 4 wk washout	DSM IV
Korgaonkar, 2014 (iSPOT)	50%	33.8	USA, UK, NL, Aus, NZ, outpatient	HDRS-17 21.0	Naïve-resistant	None, 1 wk washout	DSM IV
Williams, 2015 (iSPOT)	50%	33.8	USA, UK, NL, Aus, NZ, outpatient	HDRS-17 21.0	Naïve-resistant	None, 1 wk washout	DSM IV
v. Waarde, 2015	62%	56.6	NL, inpatient	MADRS- 10 36.5	Treatment resistant	AD 64%, AP 64%, Benz 64%	DSM IV
Patel, 2015	79%	67.4	USA, outpatient	HDRS-17 20.6	-	None, 2 wk washout	-
Redlich, 2016	61%	45.7	Germany, inpatient	HDRS-17 26.0	Treatment resistant	AD 100%, AP 74%, MS 8.7%, multiple 87%	DSM IV
Wade, 2016	74%	41.7	USA, inpatient	HDRS-17 24.3	Treatment resistant	48–72 hr washout	DSM IV
Grieve, 2016 (iSPOT)	51%	35.5		HDRS-17 22.3	Naïve-resistant	None, 1 wk washout	DSM IV
Drysdale, 2017	57%	40.4	USA, outpatient	HDRS-17 20.4	Treatment resistant	AD 60%, AP 17.7%, MS 16.9%	DSM IV
Goldstein-Piekarski, 2016 (iSPOT)	49%	32.3		HDRS-17 21.31	Naïve-resistant	None, 1 wk washout	DSM IV
Wade, 2017	-	41	USA, inpatient	-	-	-	DSM IV
Crane, 2017	59%	34.5		HDRS-17 20	Naïve-resistant	None, 3 month washout	
Goldstein-Piekarski, 2018 (iSPOT)	49%	31.7		HDRS-17 21.1	Naïve-resistant	None, 1 wk washout	DSM IV
Jiang, 2018	37%	63.8	USA, inpatient	HDRS-24 32.8	Naïve-resistant	AD 95%, AP 55%	DSM IV
Cao, 2018	58%	31.3	China, inpatient	HDRS-24	Naïve- resistant	None, ≥ 4 wk washout	DSM IV
Karim, 2018	-	65.3	USA, outpatient	MADRS 25	Naïve-resistant	-	DSM IV
Godlewska, 2018	56%	28.4	UK, -	HDRS-17	Naïve-resistant	>3 wks no med	DSM IV
Leaver, 2018	62%	41.7	USA, -	HDRS-17 24.2, MADRS 37.50	Treatment resistant	2-day washout	DSM IV
Cash, 2019	40%	34	Australia, -	MADRS	Treatment resistant	None, ≥ 4 wk washout	DSM IV
Meyer, 2019	50%	31.5	Austria, outpatient	HDRS-17 19, MADRS 27	Non-resistant, 55% AD naïve		DSM IV
Moreno-Ortega, 2019	-	60.0	USA, inpatient	HDRS-24 26.5	Treatment resistant	-	DSM IV
Sun, 2019	65%	56.3	USA, -	HDRS-17 25.6	-	-	DSM IV
Queirazza, 2019	59%	39	UK, outpatients	BDI-II 28	AD-naive	None	CIS-R

HDRS = Hamilton Depression Rating Scale, AD = antidepressants, benz = benzodiazepines, AP = antipsychotics, MS = mood-stabilizers , MADRS = Montgomery Asberg Depression Rating Scale, BDI = Beck's Depression Index, iSPOT= International Study to Predict Optimised Treatment - in Depression

Supplementary table 2: Imaging and regions of interest

Modality	Study + year	Analysis approach	ROI's / top regions	Task
**tbfMRI**				**Task**
	Marquand, 2008	PCA	FG, TG, CgC, midbrain, cerebellum, precuneus	3-back verbal memory
	Siegle, 2012	ROI, hypothesis-driven	sgACC	personal relevance rating
	Williams, 2015	ROI, hypothesis-driven	amygdala	facial emotion paradigm
	Goldstein-Piekarski, 2016	ROI, hypothesis-driven	amygdala	facial emotion paradigm
	Godlewska, 2018	ROI, hypothesis-driven, SVC	pgACC	facial expression, masked
	Karim, 2018	PCA	left iOFC, hippocampus., bilateral FG, left Cd, right PCL	facial expression / shapes
	Kraus, 2018	Gaussian kernel	right TPJ	pain anticipation
	Meyer, 2019	Context-independent FC	amPFC, dlPFC, , pCgC	n-back working memory
	Queirazza, 2019	Gaussian kernel	right amygdala, right striatum	Reversal-learning
**rsfMRI**				
	Patel, 2015*	ROI hypothesis-driven, DTM computation, PCA	dDMN, aSN	
	Van Waarde, 2015	ICA	brainstem, CB, dlPFC, dmPFC, iTC, OFC	
	Drysdale, 2015	CCA	25 regions of interest	
	Leaver, 2017	ICA	23 regions of interest	
	Wade, 2017	Automated volumetric parcellation (FreeSurfer)	CA, iTC	
	Goldstein-Piekarski, 2018	ROI, hypothesis-driven, GLM	amygdala	
	Moreno-Ortega, 2019	Multimodal parcellation	dlPFC	
	Sun, 2019	Functional connectome, CBPM	Top features: Thalamus, temporal/hippocampal/frontal gyri	
**sMRI**				
	Costafreda, 2009	Gaussian kernel	CgC, OcC, MFG	
	Nouretdinov, 2011	Gaussian kernel	CgC	
	Gong, 2011	WB, high-dimensional normalization protocol	Grey/white matter; FTC, OcC,, putamen	
	Redlich, 2016	WB, high-dimensional normalization protocol	Grey-matter integrity	
	Wade, 2016	VBM, GLM	CA, iTC	
	Cao, 2018	Automated volumetric parcellation (FreeSurfer)	CA	
	Jiang, 2018	VBM, unified segmentation	CA, left FTG, left LG, left precuneus	
**DTI**				
	Korgaonkar, 2014	DTM	CgC, ST	
	Grieve, 2016	ROI, hypothesis-driven, DTM	CgC, ST	

PCA = principal component analysis, ROI = region of interest, SVC = small-volume correction, FC = functional connectivity, DTM = diffusion tensor model, ICA = individual component analysis, CCA = canonical correlation analysis, GLM = general lenear modeling, WB = whole-brain, VBM = voxel-based morphometry, FG = frontal gyrus, TG = temoral gyrus, CgC = cingulate gyrus, sgACC = subgenual anterior cingulate cortex, pgACC = pregenual anterior cingulate cortex, iOFC = inferior orbitofrontal cortex, Cd = caudate , PCL = paracentral lobule, TPJ = tempoparietal junction, amPFC = anteriomedial prefrontal cortex, dl = dorsolateral, dDMN = dorsal default mode network, aSN = anterior salience network, iTC = enferior temporal cortex, OcC = occipital cortex, MFGL = mediofrontal gyrus, FTC = frontotemporal cortex, CA = hippocampus, ST = stria terminalis, CBPM = connection-based predictive modelling

Supplementary table 3: QUADAS quality assessment

Study	Risk of bias					Applicability concerns			
	Patient selection	*Index test*	*Reference standard*	*Flow and Timing*	*Intervention*	*Patient selection*	*Index test*	*Reference standard*	*Intervention*
Marquand, 2008	?	+	+	-	?	+	+	+	?
Costafreda, 2009	?	+	+	?	+	+	+	+	+
Costafreda 2, 2009	?	+	+	?	+	+	+	+	+
Nouretdinov, 2011	?	+	+	?	+	+	+	+	+
Gong, 2011	?	+	+	-	+	+	-	+	+
Siegle, 2012	?	+	+	-	+	+	-	+	+
Patel, 2015	?	+	+	?	-	-	- (MMSE)	+	+
Van Waarde, 2015	?	+	+	+	+	+	+	+	+
Redlich, 2016	?	+	+	+	+	+	+	+	+
Wade, 2016	?	+	+	?	+	?	+	+	+
Drysdale, 2016	?	+	+	+	+	+	+	+	+
Wade, 2017	?	+	+	?	+	?	+	+	+
Leaver, 2018	?	-	-	?	+	?	+	-	+
Cao, 2018	-	+	+	?	+	-	+	+	+
Godlewska, 2018	?	+	+	-	+	+	+	+	+
Jiang, 2018	?	+	+	+	+	- (LLD)	+	+	+
Karim, 2018	?	+	+	-	+	- (LLD)	+	+	+
Cash, 2019	?	+	+	+	+	+	+	+	+
Meyer, 2019	?	-	-	+	+	+	+	-	+
Moreno-Ortega, 2019	?	+	+	?	?	-	+	+	?
Queirazza, 2019	?	+	+	?	+	+	+	+	+
Sun, 2019	?	+	+	?	+	?	+	+	+
Korgaonkar, 2014	?	+	+	-	+	+	+	+	+
Williams, 2015	?	+	+	-	+	+	+	+	+
Goldstein-Piekarski, 2016	?	+	+	-	+	+	+	+	+
Grieve, 2018	?	+	+	-	+	+	- (non-rem)	+	+
Goldstein-Piekarski, 2018	?	+	+	-	+	+	+	+	+

? = unknown (not mentioned in article), - = high risk of bias or low applicability, + = low risk of bias or high applicability. MMSE = mini mental state examination, LLD = late-life depression, non-rem = non-remission

Supplementary table 4: Overview of grey literature

Name + year	N	Accuracy	AUC	Sensitivity	specificity	Modality	intervention
Cash, 2019	47	85-95%		-	-	rsfMRI	rTMS
Etkin, 2013	102	-		-	-	tbfMRI	SSRI
Fitzgerald, 2019	120	>85%		-	-	fMRI	rTMS
Geraci, 2014	-	83, 90%		-	-	fMRI	rTMS
Godlewska, 2017	32	75%		-	-	fMRI	SSRI
Hou, 2016	82	-	0.69, 0.71	-	-	fMRI	-
Karim, 2018	-			80	63	fMRI	SSRI
Klobl, 2019	35	-	0.73	-	-	fMRI	SSRI
Klumpp, 2019	20	80%	-	-	-	tbfMRI	CBT
Korgaonkar	157	85%		-	-	fMRI	SSRI
Kozel, 2010	13	85%		-	-	fMRI	rTMS
Langenecker, 2014	24	-	-	-	-	fMRI	SSRI
Long, 2019	59	-	-	-	-	fMRI	rTMS
Miller, 2018	31			-	-		
Narr, 2016	22	76-77%	0.75-0.80	-	-	fMRI	ECT
Nguyen, 2019	37		0.71			fMRI	bupropion
Schultz, 2018	21	88.95%	-	-	-	tbfMRI	-
Siegle	53	-	-	-	-	fMRI	SSRI
Vila-Rodriguez, 2018	62	76%-84%	0.75-0.87	-	-	fMRI	rTMS
Wade, 2015	-	-	-	-	-	fMRI	ECT
Webb, 2018	35	-	-	-	-	fMRI	CBT
Williams, 2013	101	-	-	-	-	fMRI	SSRI

N = number of participants, AUC = area-under-the curve, rsfMRI = resting-state functional MRI, rTMS = repetitive transcranial magnetic stimulation, tbfMRI = task-based fMRI, SSRI = selective-serotonin reuptake inhibitor, CBT = cognitive behavioral therapy

Supplementary figure 1: QUADAS-2 quality assessment

Applicability concerns (%)

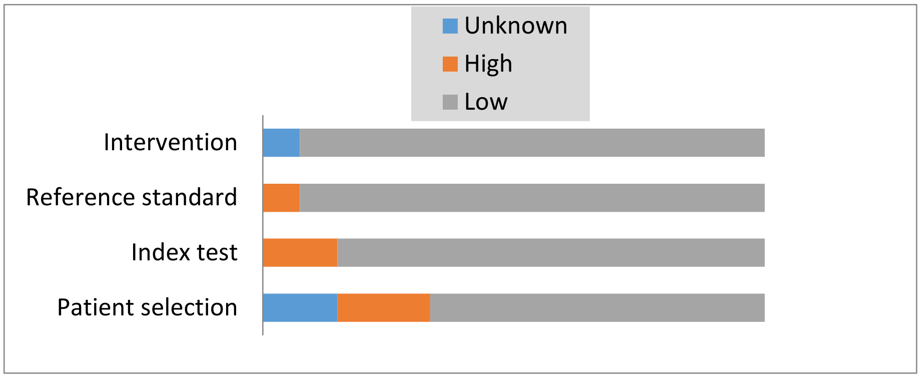

Risk of bias (%)

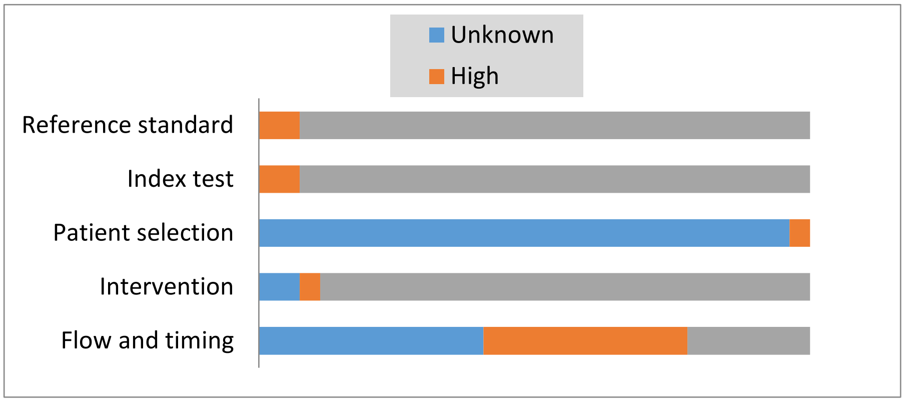

Supplementary figure 2: Deeks' test, or sample size effect:

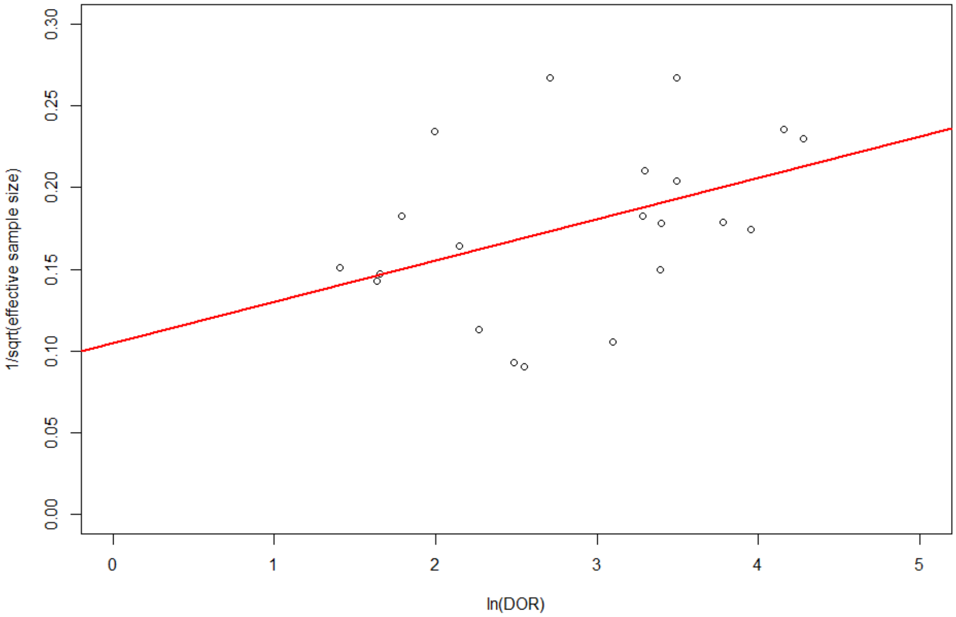

1/ √Effective Sample Size = (4xRxNR)/(R+NR) as a function of the ln Diagnostic Odds Ratio of each study. Regression equation: y = 0.11 + 0.025x, **p = 0.044** *The positive correlation between ln(DOR) and 1/√ESS indicates an negative correlation between DOR and ESS. The larger the sample size becomes, the smaller is the diagnostic accuracy. (R = responder, NR = non-responder).*

1. (NICE) NIfHaCE. Depression in adults: recognition and management (CG90). 2009.

2. Fung G, Deng Y, Zhao Q, Li Z, Qu M, Li K *et al.* Distinguishing bipolar and major depressive disorders by brain structural morphometry: a pilot study. *BMC psychiatry* 2015; **15:** 298-298.

3. Rubin-Falcone H, Zanderigo F, Thapa-Chhetry B, Lan M, Miller JM, Sublette ME *et al.* Pattern recognition of magnetic resonance imaging-based gray matter volume measurements classifies bipolar disorder and major depressive disorder. *Journal of affective disorders* 2018; **227:** 498-505.

4. Reitsma JB, Glas AS, Rutjes AW, Scholten RJ, Bossuyt PM, Zwinderman AH. Bivariate analysis of sensitivity and specificity produces informative summary measures in diagnostic reviews. *Journal of clinical epidemiology* 2005; **58**(10)**:** 982-990.

5. Macaskill P GC, Deeks JJ, Harbord RM, Takwoingi Y. *Chapter 10: Analysing and Presenting Results.* , 2010.

6. van Waarde JA, Scholte HS, van Oudheusden LJ, Verwey B, Denys D, van Wingen GA. A functional MRI marker may predict the outcome of electroconvulsive therapy in severe and treatment-resistant depression. *Mol Psychiatry* 2015; **20**(5)**:** 609-614.

7. Bossuyt P DC, Deeks J, Hyde C, Leeflang M, Scholten R. (ed). *Chapter 11:Interpreting results and drawing conclusions. In: Deeks JJ, Bossuyt PM, Gatsonis C*

(editors), Cochrane Handbook for Systematic Reviews of Diagnostic Test Accuracy Version 0.9.

2013.

8. Deeks JJ, Macaskill P, Irwig L. The performance of tests of publication bias and other sample size effects in systematic reviews of diagnostic test accuracy was assessed. *Journal of clinical epidemiology* 2005; **58**(9)**:** 882-893.

9. van Enst WA, Ochodo E, Scholten RJPM, Hooft L, Leeflang MM. Investigation of publication bias in meta-analyses of diagnostic test accuracy: a meta-epidemiological study. *BMC medical research methodology* 2014; **14:** 70-70.

10. Redlich R, Opel N, Grotegerd D, Dohm K, Zaremba D, Burger C *et al.* Prediction of Individual Response to Electroconvulsive Therapy via Machine Learning on Structural Magnetic Resonance Imaging Data. *JAMA psychiatry* 2016; **73**(6)**:** 557-564.

11. Jiang R, Abbott CC, Jiang T, Du Y, Espinoza R, Narr KL *et al.* SMRI Biomarkers Predict Electroconvulsive Treatment Outcomes: Accuracy with Independent Data Sets. *Neuropsychopharmacology* 2017; **43:** 1078.
